# The role of network connectivity on epileptiform activity

**DOI:** 10.1038/s41598-021-00283-w

**Published:** 2021-10-21

**Authors:** G. Giacopelli, D. Tegolo, M. Migliore

**Affiliations:** 1 Department of Mathematics and Informatics, University of Palermo, Palermo, Italy; 2 Institute of Biophysics, National Research Council, Palermo, Italy

**Keywords:** Computational biophysics, Network models, Applied mathematics, Epilepsy

## Abstract

A number of potentially important mechanisms have been identified as key players to generate epileptiform activity, such as genetic mutations, activity-dependent alteration of synaptic functions, and functional network reorganization at the macroscopic level. Here we study how network connectivity at cellular level can affect the onset of epileptiform activity, using computational model networks with different wiring properties. The model suggests that networks connected as in real brain circuits are more resistant to generate seizure-like activity. The results suggest new experimentally testable predictions on the cellular network connectivity in epileptic individuals, and highlight the importance of using the appropriate network connectivity to investigate epileptiform activity with computational models.

## Introduction

Epilepsy is a relatively common and widespread brain disease affecting people of all ages^1^. According to the World Health Organization, this disease affects nearly 50 million people^2^. For this reason, there are extensive experimental and theoretical efforts attempting to figure out the mechanisms underlying the onset and propagation of the plethora of abnormal (and transient) brain electrical activity caused by this disease. A better understanding of the involved mechanisms can facilitate the development of therapeutic solutions. A number of potentially important mechanisms have been identified as key players, such as genetic variations^3^ often associated with ion channels mutations^4^, alterations of synaptic function^5^, and network connectivity^6,7^. However, current technical problems make the study of their specific contribution very difficult to investigate experimentally. From this point of view, computational models^8,9^ can be a very convenient approach to identify the relative role and importance in generating seizures and, more generally, epileptiform activity.

Here we study, in more details, the role of network connectivity at cellular level, by exploiting a new mathematical framework^10,11^ that can create networks with connectivity properties similar to those observed in real brain networks, we studied how network connectivity can affect the onset of epileptiform activity. The model suggests that networks connected as in real brain circuits are more resistant to generate seizure-like activity. The results suggest new experimentally testable predictions and highlight the importance of using the appropriate network connectivity to investigate epileptiform activity with computational models.

## Results

**Figure 1:**
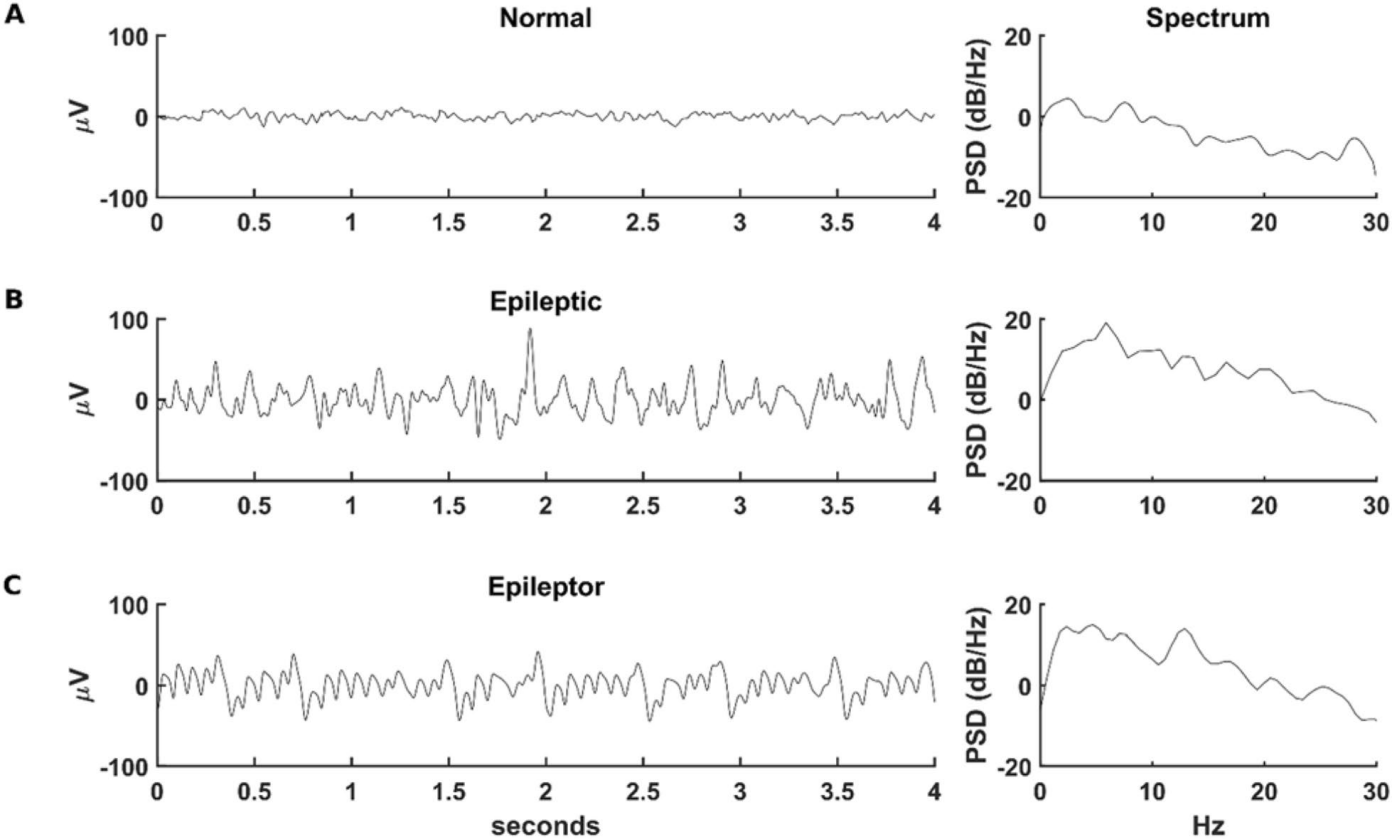
Comparison of experimental and model traces. (**A**) typical EEG trace under control conditions (left, from^12^), and the corresponding Welch spectrum (Right); (**B**) typical EEG trace during epileptiform activity (from^13^); (**C**) time course of the variable corresponding to the membrane voltage during a simulation of an Epileptor^15^ with parameters: *a* = 1, *b* = 3, *c* = 1, *d* = 5, *I*_*ext1*_ = 3.1, m = 0, *a*_2_ = 6, *τ*_2_ = 10, *I*_*ext2*_ = 0.4, *γ* = 0.01 and a white noise of amplitude 0.25. The adimensional model has been converted in a dimensional one using the time scale constant 0.0167 s and the voltage scale constant 25 µV.

In Fig. 1A we report a typical normal EEG recording^12^, and in Fig. 1B a trace exhibiting epileptiform activity from a public database^13^, together with the corresponding Welch spectrograms^14^. The epileptic trace shows the characteristic high-amplitude population spikes and a significant increase in the spectral density in the low frequency range (compare right plots of Fig. 1A,B). A typical model trace, obtained from the latest Epileptor version^15^, and the corresponding spectral density is shown in Fig. 1C. In the example traces shown in Fig. 1, the mean amplitude of the voltage was 6.9, 29.9, and 30.8 µV for the normal, epileptic, and Epileptor trace, respectively. The spectral analysis of the EEG signals, combined with other features^16^, has a key role in seizure detection^17^ and in building stable and accurate seizure detecting algorithms^18^. Another immediate and obvious measure of epileptic activity is the mean signal amplitude. However, it may be less reliable that spectral analysis because it can be affected by a number of artifacts, such as detectors’ sensitivity, external noise, experimental conditions, etc. Since our simulations are not affected by these problems, in the rest of the paper we will define a trace as exhibiting an epileptiform activity when the average membrane potential is above 18.4 µV, half-way between the values calculated from the normal and epileptic experimental traces.

In order to investigate the role of network connectivity in the onset of epileptiform activity, we considered two aspects: (1) the type of connectivity (exponential or convolutive), (2) the spatial arrangement of the individual neurons in a volume, shaped as a column (like in the cortex) or as a slice (like in the in vitro hippocampal preparation). The rationale for this choice was that we were interested in studying the response of networks connected using a convolutive model, and compare the results obtained with networks implemented using the widely used ER model^19^ in which neurons are connected with a fixed connection probability. We think that this is an important point. A convolutive model, introduced in^10^, is based on the observation that the degree distributions of real brain networks do not follow neither an ER nor a power law model, but a mix of both^20^. Based on rigorous mathematical considerations, a convolutive model is constructed by first subdividing the network volume in blocks and then creating the connections inside the blocks with a power law scheme (short range connections) and the connections between blocks with an ER scheme (long range connections). It can be mathematically proved that such a procedure generates a network matching the experimentally observed degree distributions^10,11^. It can thus provide a better insight into the network mechanisms responsible for the onset of epileptiform activity in the real brain.

**Figure 2:**
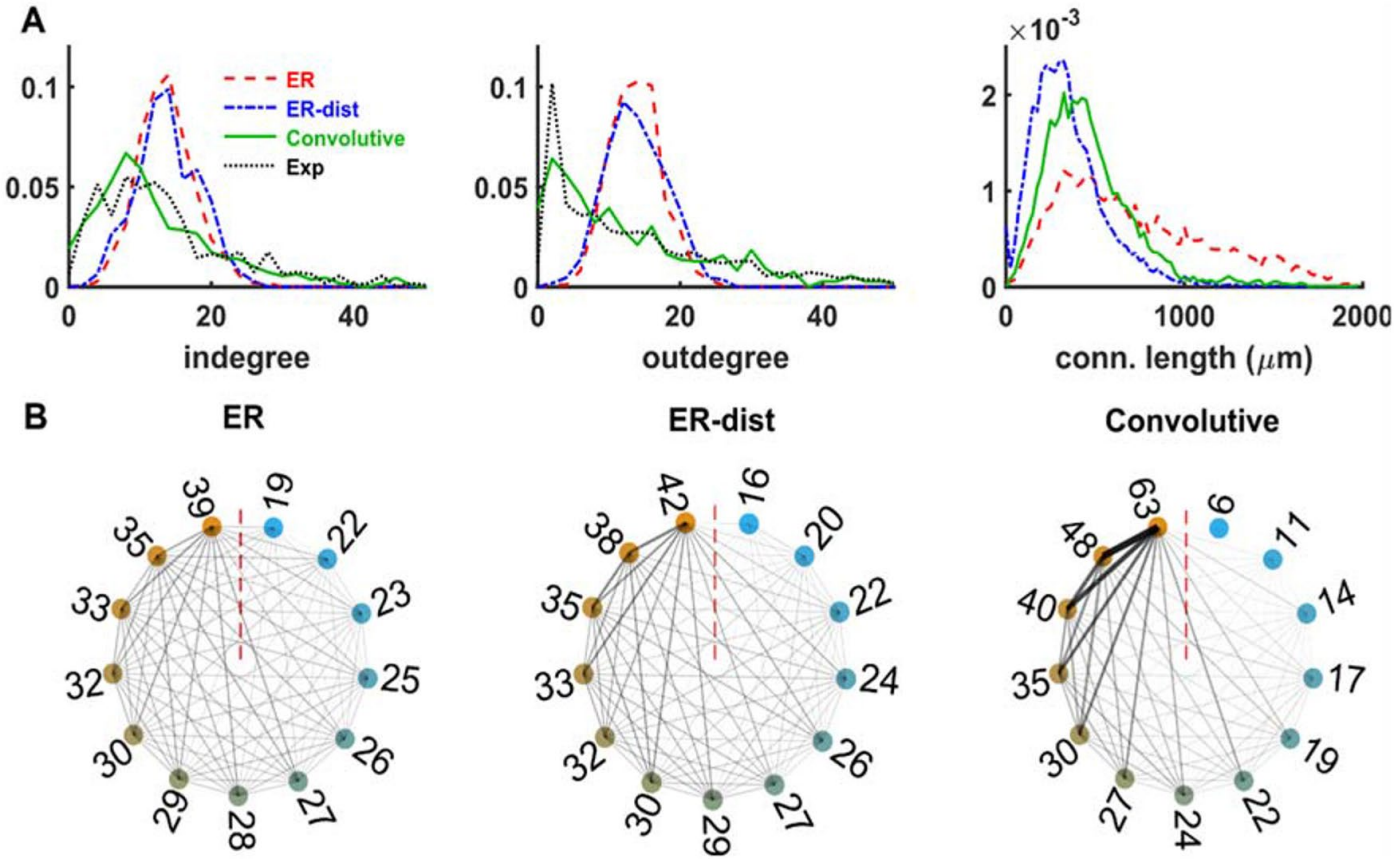
Neurons distributed in a thick rectangular volume. (**A**) In-degree distribution (left), out-degree distribution (middle) and connection length distribution (right) for networks of Epileptors connected as an ER model (red), ER with distance (blue) and using a convolutive model (green) fitted to experimental *C. elegans* data (black, connection length was not available). (**B**) Circular graph representation of connectivity for the ER model (left), the ER-dist model (middle) and Convolutive model (right). See the main text for an explanation of how they were constructed. The lines thickness and transparency are proportional to the number of connections between any two given groups.

In Fig. 2A we plot the degree and connection length distributions of the networks implemented by uniformly distributing the 550 Epileptors in a rectangular volume, connected in three different ways: (1) following an ER model, ignoring the distance among neurons (Fig. 2A, red traces), and with a fixed connection probability of *p* = 0.026, corresponding to the average connection probability of a *C. elegans* brain, (2) following an ER model but with a connection probability depending on the distance between neurons as *p*(*d*) = *Ae*^−*Bd*^ (as in^21^), where *d* is the distance between two nodes, *A* = 0.2, and *B* = 0.004 (Fig. 2A blue traces), and (3) using a convolutive model (Fig. 2, green traces) fitting a *C. elegans* degree distributions^22^, with two blocks and parameters, *δ* = 1.5, *Ek* = 1 and *η* = 3 (see “Methods”). The degree distributions using the ER models (Fig. 2A red and blue traces) were very similar to each other, even if their connection length distributions were very different (compare red and blue lines in the right panel of Fig. 2A). This suggests that modifying the connection probability by including information on the distance between neurons does not significantly change the degree distributions. The qualitative difference, between the ER models and the convolutive model, in the connectivity among neurons, can be better appreciated using a graphical representation as in Fig. 2B, where we plot a circular graph representing the connectivity of different networks. The graphs were constructed by using clockwise-placed bins representing increasing total degrees (calculated as the sum of in- and out-degree); neurons were then assigned to each bin according to their total degree, and bins were connected with a line that was progressively thicker and less transparent as a function of the average number of connections between any two given groups. Following this scheme, thin faint lines represent poorly connected groups, thick black lines highlight strongly connected groups. Note the overall relatively light and uniform connectivity in ER-type networks (Fig. 2B, left and middle plots), in striking contrast with the overall bolder connectivity of the convolutive model network, preferentially connecting groups of higher degrees.

**Figure 3:**
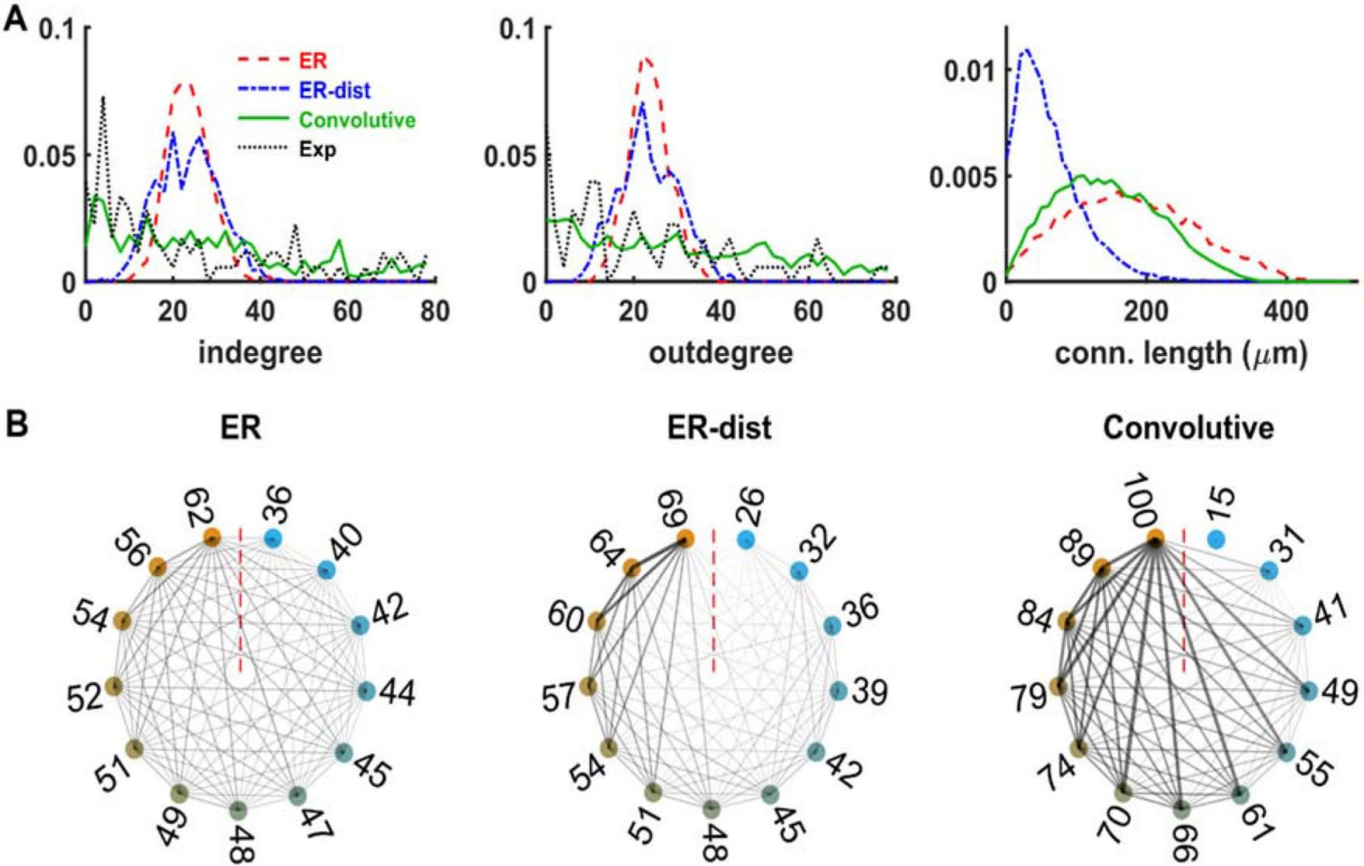
Neurons distributed in a thin rectangular volume. (**A**) In-degree distribution (left), out-degree distribution (middle) and connection length distribution (right) for networks of Epileptors connected as an ER model (red), ER with distance (blue), and using a convolutive model (green), connected as observed in an experimental hippocampal slice (black^23^), implemented with four blocks and two sub-blocks containing 1% and 99% of the neurons, respectively, and with parameters *δ* = 0.25, *Ek* = 0.1 and *η* = 3. (**B**) Circular graph representation of connectivity for the ER model (left), the ER-dist model (middle) and Convolutive model (right). See the main text for an explanation of how they were constructed. The lines thickness and transparency are proportional to the number of connections between any two given groups.

The differences were more evident for networks of neurons distributed in a volume shaped as a slice, i.e. a rectangular volume of 400 × 300 × 10 μm, with an average connection probability (*p* = 0.0438) consistent with that observed in a hippocampal slice^23^, as shown in Fig. 3. Note that these networks have a higher neuron density and connection probability, with respect to those implemented in a rectangular box. Compared to the ER model (Fig. 3A, red), the ER-dist model (Fig. 3A, blue) now exhibited a different connectivity, a direct consequence of the very different connection length distribution (Fig. 3A, right). Both models, created with parameters *A* = 0.7 and *B* = 0.025, were rather different from the results obtained with the convolutive model (Fig. 3A, green) obtained by fitting (*p* > 0.05) the experimentally observed degree distributions (Fig. 3A, black) of a hippocampal slice. The connection length distribution reflected the very different shape and size of the volume in this case (note the different scale for the distance axis in Figs. 2A and 3A), with a proportion of longer connections for the convolutive model more similar to the ER model built without distance-dependent connectivity. The circular graph representation in Fig. 3B, reinforces the contrast between the rather uniform connectivity of neurons belonging to an ER network and the clear predominance of connections between hub neurons for the convolutive model. These results highlight the large overall structural differences between networks with a realistic connectivity and those based on a fixed connection probability, independently from the network shape and size. Although ER networks have been shown to be able to reproduce selected experimentally observed firing patterns under physiological conditions, provided that different neuron populations have different connection probabilities^24^, it has also been shown that they are more likely to synchronize, with respect to power-law networks^25^. Furthermore, from a more general point of view, ER networks respond to an input in a way that is significantly different from that obtained from a convolutive network^20^. Taken together, these results, as we will see in the following paragraphs, suggest that a network connected as in a real brain can be significantly more resistant to generate an epileptiform activity, with respect to networks with a fixed connection probability. Considering that seizures can significantly change the functional brain connectivity^6^, it can be argued that seizures, when they begin to occur more and more often in a normal brain during the progression of the disease, may change the network’s connectivity to make it more similar to a random network.

### Networks with realistic connectivity are less prone to generate epileptiform activity

**Figure 4:**
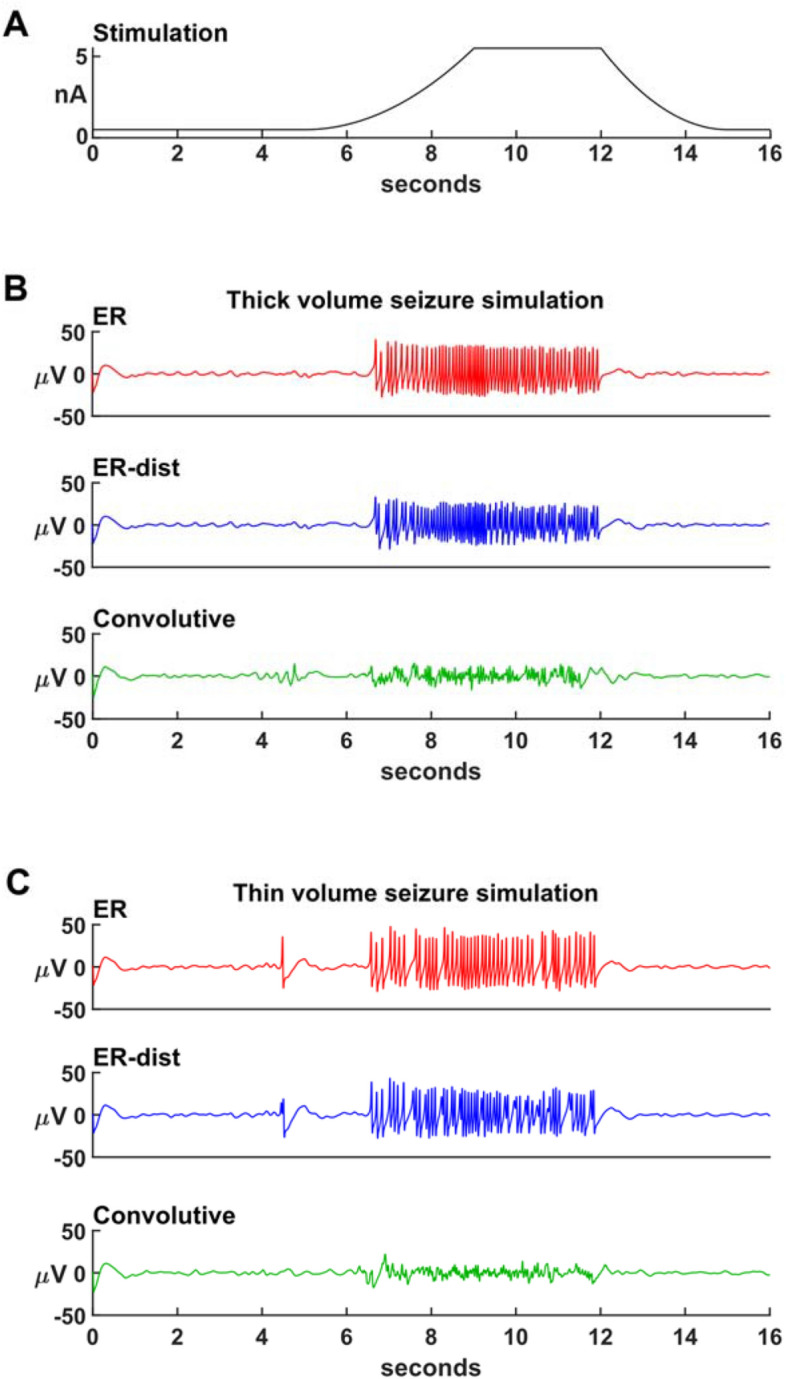
Networks with realistic connectivity are less prone to generate epileptiform activity. (**A**) Maximum input current, in the center of the network, as a function of time; (**B**) simulation of epileptiform activity in networks of Epileptors distributed in a thick rectangular volume and connected in different ways, with low inhibitory interaction (*n* = 0.1). (**C**) Same as in (**B**) but with neurons distributed into a thin rectangular volume.

To test the response of our networks to an external input, we carried out a series of 16 s simulations during which we activated, on top of the background activity, an external current, spatially modulated as described in the Simulations section, with *I*_*max*_ = 5 nA and the time course shown in Fig. 4A. In Fig. 4B,C we plot the response of the networks, in terms of average membrane potential over all neurons, under different conditions of spatial distributions and connectivity rules. All networks received the same input. Before the stimulus onset, the activity in all cases appeared to have a normal behavior, with occasional and short-lived spikes of higher intensity, more noticeable for the ER models in thin volume. As the external input increased, all ER model networks (with or without distance dependent connectivity) started to have an epileptic behavior, with an average value in the 5–15 s time window of 46.6, 34.6, 47.3 and 37.1 µV, for the ER and ER-dist in thick volume and for the ER and ER-dist in thin volume, respectively. Networks connected as in real brain networks (Conv. Model in Fig. 4B,C), had an average value of 9.9 and 7 µV, for thick and thin volumes, respectively, very close to the value obtained from normal traces. These results suggest that the structural connectivity of real brain networks is more resistant to enter into an epileptic state.

### Realistic connectivity robustly protects from the consequences of strong inputs

**Figure 5:**
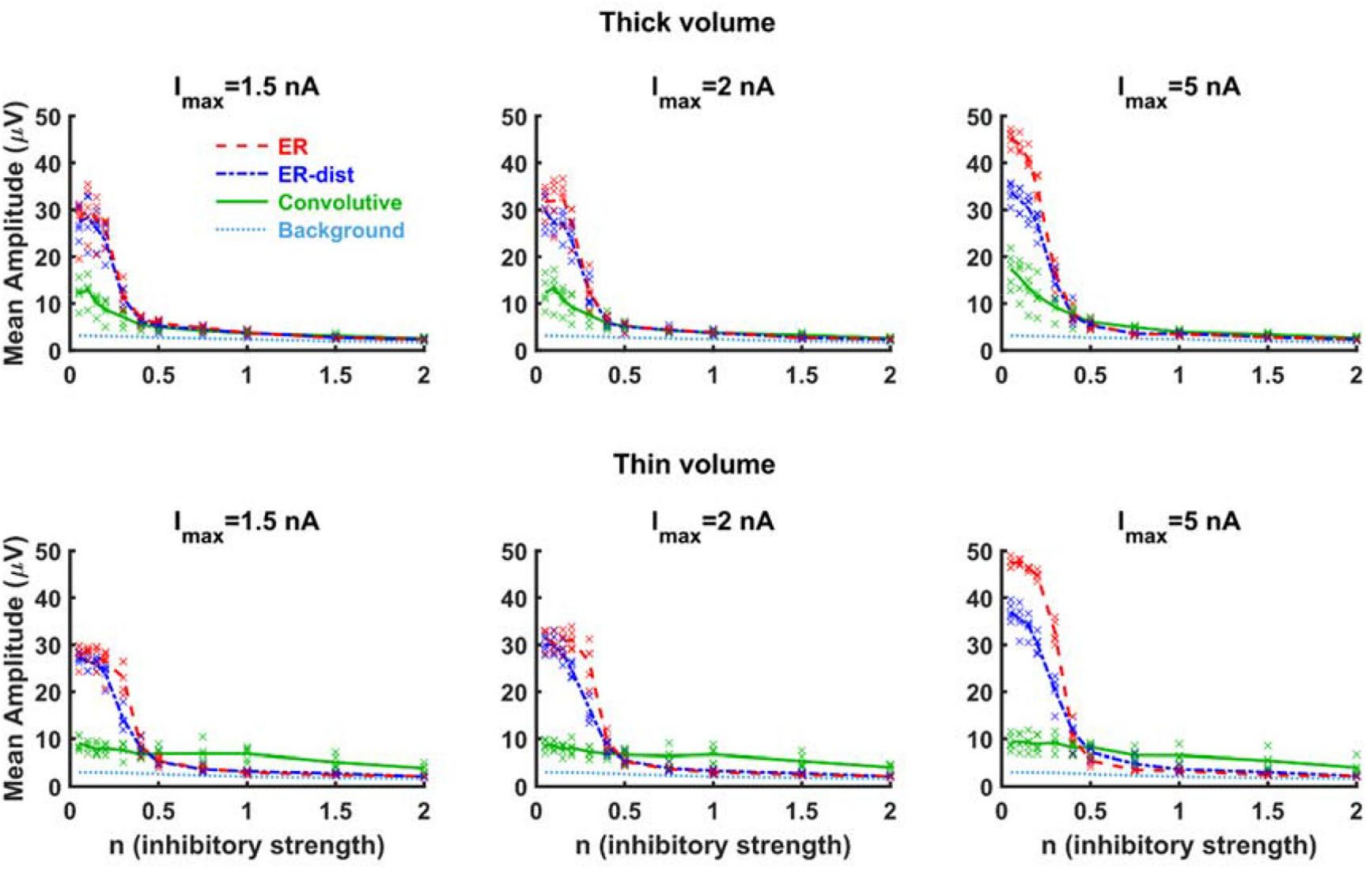
Realistic connectivity robustly protects from the consequences of strong inputs. Average membrane potential of the different models calculated, as a function of the inhibition, over the 10 s duration of an external input of different maximum strengths, *I*_*max*_. For any given value of *I*_*max*_ and *n*, symbols represent the average membrane potential obtained from five network instances. Lines represent their average value.

In addition to the input strength, the amount of inhibition generated by network activity is a well-known modulatory mechanism for epileptiform activity. Thus, we explored this aspect by carrying out a series of simulations in which we progressively increased the parameter *n*, regulating the inhibitory interaction among neurons. Simulations were performed for three values for *I*_*max*_ and a discrete set of *n* values (0.05, 0.1, 0.15, 0.2, 0.3, 0.4, 0.5, 0.75, 1.0, 1.5, 2.0). For each combination, we simulated five network instances. In Fig. 5, we show the mean amplitude during the stimulation for each model. As expected, increasing the inhibition will eventually reduce the average amplitude of the signal. However, large values of *n* should not be considered physiological because, under these conditions, the network will strongly inhibit also physiological signals. If we focus on the lower range of *n* values (e.g. below 0.5) it can be observed that simulations using the convolutive model have a mean amplitude lower than exponential models at all currents (Fig. 5, top panels). It should also be noted that, for high input currents, ER distance models have lower mean amplitudes than ER models, pointing out the importance of spatial information in building networks models. The same overall trend can be observed in the bottom panels of Fig. 5, for a thin volume model. In this case, it should be noted that the higher neuron density and connection probability, with respect to the rectangular box, results in a higher number of (both excitatory and inhibitory) hubs that prevents the onset of an epileptiform activity even for small inhibitory strength. These results suggest that a realistic connectivity, implemented though a convolutive model, robustly protects from the onset of epileptiform activity during a wide range of strong external inputs.

### Spike-wave complexes

**Figure 6:**
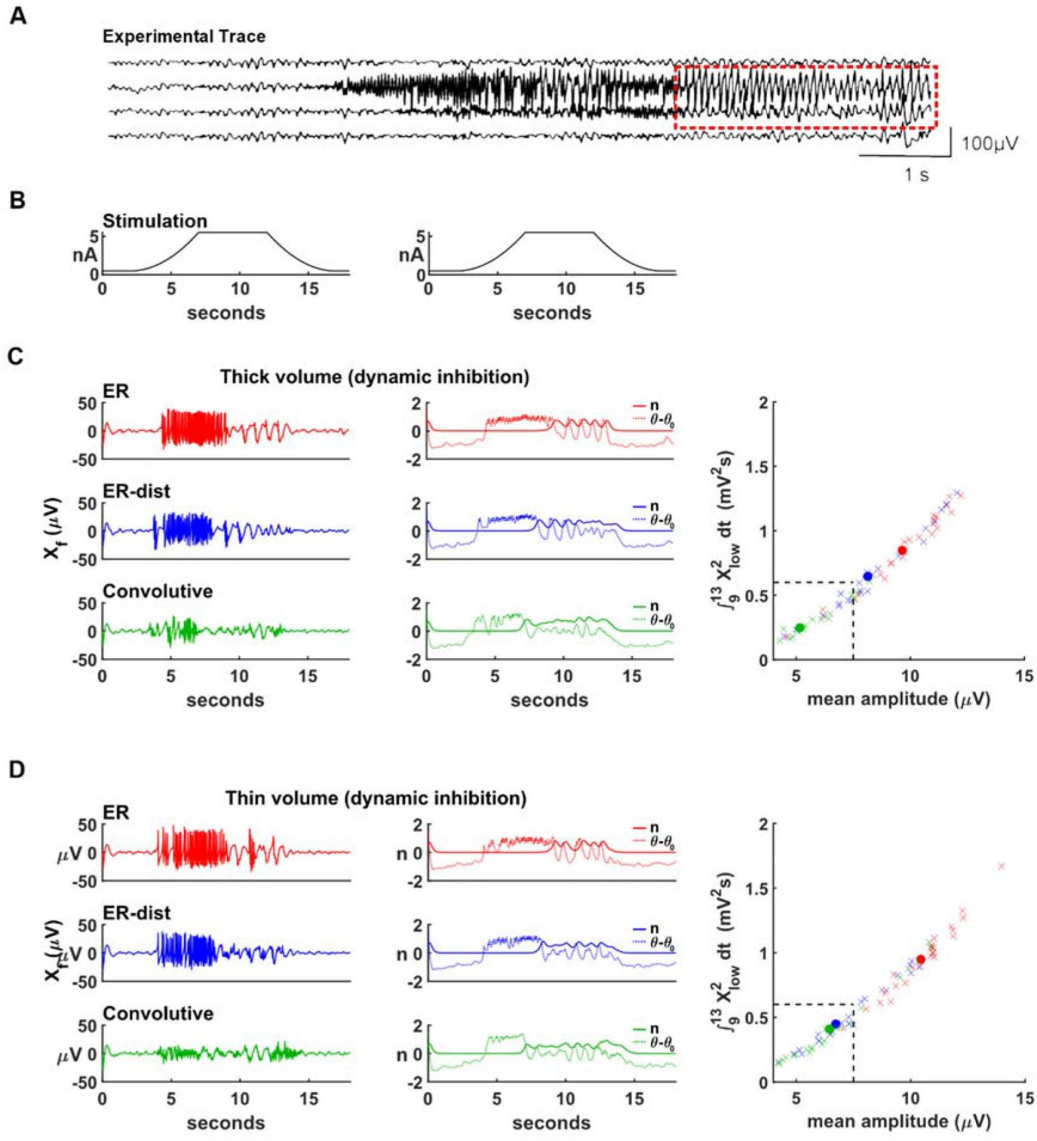
Dynamic inhibition can underlie spike-wave complexes. (**A**) Experimental EEG recording showing a period of spike-wave complexes (from^26^), indicated by the red outline; (**B**) maximum input current, in the center of the network, as a function of time; (**C**) Typical average membrane potential, *X*_*f*_, for neurons distributed in a thick volume (left), time course of the variables *n* and θ, associated with the slow inhibitory interaction (middle), the integral ${\int }_{9}^{13}{X}_{low}^{2}dt$ as a function of the mean amplitude, for all the 25 simulations carried out for each model using different random number seeds (right); red, blue, and green closed circles indicate the average value for each model; the dotted line indicates the grand average of the integral, calculated from all simulations. (**D**) Same as in (**C**) but for neurons distributed in a thin volume.

Epileptiform activity includes spike-wave complexes^26^, which are identified as EEG portions with variable amplitude at around 3 Hz frequency^27^. A typical spike-wave complex period is shown in Fig. 6A. With our Epileptor networks, we found that they can be modeled by assuming a dynamic modulation of the inhibitory strength, supporting the idea^28^ that they can emerge from a mutual inhibition–rebound interaction. This corresponds to the physiological plausible assumption that the inhibitory interaction among neurons follows network activity (in terms of average membrane potential), generating a dynamic level of inhibitory response. We implemented this effect by modulating the value of *n*, the variable responsible for the inhibitory interaction among Epileptors, as:

1 $$\begin{array}{c}\tau \frac{dn}{dt}= \rho \left(\theta -{\theta }_{0}\right) \left({n}_{0}-n\right) n\end{array}$$

2 $$\begin{array}{c}\tau \frac{d\theta }{dt}= {\rho }_{\theta } \left(\frac{X\left(t\right)}{\nu }-\theta \right)\end{array}$$

where *τ* = 0.0167 s, *ν* = 20 µV, *n*_0_ = 2, *ρ* = 0.05, *ρ*_*θ*_ = 0.15 and *θ*_0_ = {− 0.9; − 1} for the thick or the thin volume, respectively. These equations result in a logistic behavior for *n* (for θ constant) with a plateau value of *n*_*0*_. The variable θ slowly converges to the value *X/ν* and modulates, through the $\left(\theta -{\theta }_{0}\right)$ term, the inhibitory action of *n*.

For these simulations, the input stimulus time course is shown in Fig. 6B, and the time course of the average membrane potential, representative of the behavior observed for the different models in a thick volume network, is reported in Fig. 6C (left plot). To better characterize spike waves activity using a quantitative measure, we evaluated the integral ${\int }_{9}^{13}{X}_{low}^{2}dt$ for all the 25 simulations carried out using different random number seeds, with *X*_*low*_ calculated as the average membrane potential filtered through a 333 ms (i.e. 3 Hz) moving average window. The values were then plotted as a function of their mean amplitude (Fig. 6C, right plot). The grand average value of the integral, calculated from all the 75 simulations (25 for each model) that were carried out, was used as a threshold to define a spike wave period as an epileptic activity. This value is indicated by a dotted line in the right panel of Fig. 6C.

Spike-wave complexes were clearly evident in the ER models (Fig. 6C, left), following a period of a seizure-like pattern. The average values during the entire stimulation period (3–17 s), were 32.2 and 20.9 µV. Spike-wave complexes were also present for the convolutive model (Fig. 6C, green trace) but they were of much smaller amplitude, and the overall average activity during the stimulation period was within the range for a normal activity (9.4 µV).

The time course of *n* and *θ*–*θ*_0_ (Fig. 6C, middle plot), can give some hints on the complex system dynamics responsible for the emergence of spike waves. It reveals that the interaction between these two components (see Eqs. (1) and (2)), and their relatively slow dynamics, have a key role in initiating a spike waves period. During the high-frequency oscillations, the *θ*–*θ*_0_ increases, until it eventually begins to significantly affect the sigmoidal *n* dynamics (through Eq. 2). It is at this point that spike-wave activity begins. While for the more homogeneous ER networks this interaction can more easily promote large scale oscillations, the more structured convolutive network was more resilient to activate this activity. This latter aspect explains why in networks of neurons distributed in a thin volume (Fig. 6D), where the overall connectivity is stronger and more compact, spike-wave complexes fall below threshold also for the ER-dist model (Fig. 6D, green traces). Taken together, these results suggest that structural network connectivity can have an important role in the emergence of spike-wave complexes of pathological amplitudes.

## Discussion

The results presented in this paper point to a paramount role for network connectivity in modulating the onset of epileptiform activity during strong localized inputs, which can arise during a variety of physiological brain states or cognitive activities^29^. The relevance of these findings can be discussed in the context of both modeling and experiments.

From the modeling point of view, it should be stressed that large-scale neuronal network models, aiming at studying the onset and propagation of epileptic activity, so far have been almost exclusively implemented using all-to-all or fixed connection probabilities. However, these connectivity rules do not represent the real connectivity experimentally observed in brain networks. We have previously shown^20^ how this difference can generate significant variations in the response of a network to a given input. Here, using a recently published mathematical framework, able to create convolutive model networks reproducing experimental findings^10,11^, we have shown that networks connected in the same way as real systems are less prone to generate epileptiform activity. Furthermore, the model’s suggestion that spike-wave complexes can be generated by a dynamic inhibitory interaction, also points to a possible improvement on how to reproduce this specific experimental feature in a model. These results thus suggest that network models should be built using physiologically plausible connectivity rules, rather than fixed connection probabilities. This will make it easier to interpret experimental findings in terms of model parameters and it will allow making more precise and successful, experimentally testable, predictions.

From the experimental point of view, our results suggest that epileptiform activity can arise from an epileptogenic process during which a network (or a significant part of it) is progressively transformed from normal to epileptic through an ictogenetic mechanism that modifies the connectivity distribution functions (i.e. in/out-degrees and connection length) from a convolutive to an exponential scheme. This process is equivalent to selectively lose hub neurons, which makes the network less robust and more likely to generate epileptiform activity in the presence of strong inputs. There are a number of experimental findings indirectly supporting this result (reviewed in^1^), which may be the consequence of pathogenetic mechanisms altering synaptic plasticity and axonal sprouting. Even if pathological situations, including epilepsy, can activate important compensatory mechanisms of network reorganization involving the upregulation or neural stem cell activity^30^, the process of hubs formation and integration into a network is considerably long, and requires a protracted period of consolidation starting at the prenatal stage and extending into late adolescence^31^. Losing hubs during adulthood may thus have rather dramatic and long-lasting consequences^32^.

Finally, the model suggests two specific experimentally testable predictions. The first one is that epileptic tissue should have a distinctly different cellular connectivity, with respect to normal tissue: there should be less hub neurons in the epileptic tissue. The second one is that spike-wave complexes can be modulated by a dynamic inhibition much higher than during normal activity. The role of inhibition in controlling epileptic activity is well known, and its generalized increase is the classic strategy followed by antiepileptic drugs^33^. However, this action has a wide range of negative collateral effects^34^. The model suggests that better results could be obtained by selectively increasing inhibition only when and where needed, i.e. in highly active regions. Under this condition, synapses are presumably activated at a higher rate. Pharmacological applications, acting above a threshold activation level on the biochemical processes underlying short-term facilitation of inhibitory synapses, could increase the amount of signal summation only during high-frequency synaptic activation in localized regions, generating less collateral effects in the rest of the brain. From this point of view, there are already experimental indications that specific proteins or compounds can regulate synaptic transmission, such as synapsins, which can regulate GABA release^35^, and antiepileptic drugs can alter short-term plasticity^36^. An extremely promising line of action is to selectively control the activity of specific neuronal populations, and it has been shown that the use of optogenetic and designer receptor technologies for this purpose^37^ can have a strong impact on reducing or avoiding seizures onset.

## Methods

### Single neuron model

The first step in building a network is to decide on the type of individual neurons composing it. We decided to start from the Epileptor (from now on EP)^38^, which has been demonstrated to be able to reproduce epileptiform activity at the macroscale level, and it has been successfully used to simulate the possible scenarios after surgery in patients with drug-resistant seizures^15^. The original model uses six differential equations to represent the activity of a population of Hindmarsh–Rose (HR) neurons^39^, under a mean-field approximation (defined as the average of the membrane potential associated to neurons)^40^. In order to more easily implement different connectivity rules, we modified this model by using independent equations for the excitatory and inhibitory neurons, and by adding units to the original adimensional implementation. The membrane potential of an excitatory neuron, *x*_*Ei*_, was thus defined as:

3 $$\begin{array}{c}\tau \frac{d{x}_{Ei}}{dt}={\nu y}_{Ei}-a \frac{ {{x}_{Ei}}^{3}}{{\nu }^{2}}+b\frac{{ {x}_{Ei}}^{2}}{\nu }-{\nu z}_{Ei}+K \frac{\sum_{k=1}^{{N}_{E}}{{A}^{EE}}_{ik}\left({x}_{Ek}-{x}_{Ei}\right)}{{N}_{E}}+\\ -n K\frac{\sum_{k=1}^{{N}_{I}}{{A}^{EI}}_{ik}\left({x}_{Ik}-{x}_{Ei}\right)}{{N}_{I}}+\sigma I\left(t, m\right)+\omega \left(t\right)\end{array}$$

4 $$\begin{array}{c}\tau \frac{d{y}_{Ei}}{dt}=c-d \frac{{{x}_{Ei}}^{2}}{{\nu }^{2}}-{y}_{Ei}\end{array}$$

5 $$\begin{array}{c}\tau \frac{d{z}_{Ei}}{dt}=r \left(s \frac{{x}_{Ei}-{x}_{0}}{\nu }-{z}_{Ei}\right)\end{array}$$

Analogously, the equations for an inhibitory neuron *j* were defined as:

6 $$\begin{array}{c}\tau \frac{d{x}_{Ij}}{dt}={\nu y}_{Ij}-a \frac{{{x}_{Ij}}^{3}}{{\nu }^{2}}+b\frac{{ {x}_{Ij}}^{2}}{\nu }-{\nu z}_{Ij}+K \frac{\sum_{k=1}^{{N}_{E}}{{A}^{IE}}_{jk}\left({x}_{Ek}-{x}_{Ij}\right)}{{N}_{E}}+\sigma I\left(t, m\right)+\omega \left(t\right)\end{array}$$

7 $$\begin{array}{c}\tau \frac{d{y}_{Ij}}{dt}=c-d \frac{{{x}_{Ij}}^{2}}{{\nu }^{2}}-{y}_{Ij}\end{array}$$

8 $$\begin{array}{c}\tau \frac{d{z}_{Ij}}{dt}=r \left(s \frac{{x}_{Ij}-{x}_{0}}{\nu }-{z}_{Ij}\right)\end{array}$$

where *x* is the membrane potential (in µV) of neuron *x*, *y* and *z* are the fast–slow variables of the original EP model, *N*_*E*_ and *N*_*I*_ are the number of excitatory and inhibitory neurons, respectively, the parameters *a* = 1, *b* = 3, *c* = 1, *d* = 5, *s* = 4, *r* = 0.003, *x*_0_ = − 1.6 and *K* = 150 define the fast–slow interaction, and n is the inhibitory strength. The matrices *A*_*EE*_*, A*_*EI*_ and *A*_*IE*_ are the adjacency matrices for each type of connection (Excitatory–Excitatory, Excitatory–Inhibitory and Inhibitory–Excitatory; Inhibitory–Inhibitory connections were ignored, as in^38^). The variables *τ* = 0.0167 s, *ν* = 20 µV and *σ* = 1 µV/nA allowed us to introduce units to the model. The function *ω*(*t*) is a noise variable and *I*(*t*,*m*) is the time- and space-dependent external current.

### Theoretical background for network connectivity

Neurons’ connectivity can be encapsulated in the concept of spatial graphs^11^. A spatial graph *G* is an abstract entity composed by a set of vertices *V* (in our case, the neuron somas), a set of edges *E* ⊆ *V* × *V* (in our case, the synaptic connections), and a set of positions (in our case, the spatial coordinates of the somas). Each vertex is associated with in-degrees (the number of incoming connections) and out-degrees (the number of the outgoing connections). The set of degrees is described by the probability density function of their distribution. Finally, a connection length distribution can also be associated to a network with spatially distributed nodes.

Neuronal networks are very often connected following either a trivial all-to-all rule or according to fixed probabilistic rules based on two main graph-theoretical models: power law or exponential. Power law models have a heavy tail behavior in the degree distributions^41^, whereas exponential models (^42^; since now ER) use a fixed connection probability rule that generates an exponential decay of the degree distributions around a central value. Real brain networks are not connected all-to-all, but exhibit a mix of exponential and power law connectivity models: an exponential behavior for low degrees, and a power law behavior for high degrees.

We have recently mathematically demonstrated how an appropriate convolution of these models can quantitatively reproduce the degree distributions observed in a real brain network^10,11^. The key idea of the algorithm behind the theory is to split a network in blocks, and use a heavy tail connectivity inside the blocks and an exponential connectivity between blocks. In detail, assuming that Ω_A_ and Ω_B_ are two spatial regions with *N*_*A*_ and *N*_*B*_ neurons, respectively, within each region a power law tail connectivity is created using a growing network algorithm^43^ with a cost function defined as

9 $$\begin{array}{c}{C}_{hj}=\frac{\delta \left({d}_{hj}+{S}_{N}\eta {r}_{j}\right)}{{S}_{F}}+{\lambda }_{j}\end{array}$$

where *δ* and *η* are parameters of the model, *d*_*hj*_ is the square of the Euclidean distance between nodes *h* and *j*, *λ*_*j*_ is a graph theoretical measure of centrality, *r*_*j*_ is a uniform random number in [0, 1], *S*_*N*_ = 200 µm^2^ and *S*_*F*_ = 1 µm^2^. If *f*^*I*^_*k*_ and *f*^*O*^_*k*_ are the limit distributions of this process, assuming an ER connectivity between blocks, and defining the graph *C* as the union between *A* and *B*, it can be mathematically proved^10,11^ that

10 $$\begin{array}{c}P\left({D}_{C}^{I}=k\right)=\frac{{N}_{A}}{{N}_{A}+{N}_{B}}[{f}_{q}^{I}*{B}_{p}^{{N}_{B}}\left(q\right){]}_{k}+\frac{{N}_{B}}{{N}_{A}+{N}_{B}}[{f}_{q}^{I}*{B}_{p}^{{N}_{A}}\left(q\right){]}_{k}\end{array}$$

where *P*(*D*^*I*^_*C*_ = *k*) is the in-degree distribution of graph *C*, *B*_*p*_^*N*^(*k*) is the binomial distribution and * is the convolution operator defined between two discrete distributions *h*_*k*_ and *g*_*k*_ as

11 $$\begin{array}{c}[{h}_{q}*{g}_{q}{]}_{k}= \sum_{q=-\infty }^{+\infty }{h}_{k-q}{g}_{q}.\end{array}$$

The out-degree distribution is described in the same way. A web application allowing to create an arbitrary network with the desired distributions is available in the live papers section of the Human Brain Project (HBP) (https://humanbrainproject.github.io/hbp-bsp-live-papers/index.html), and the python code with model and simulation files can be downloaded from ModelDB (https://senselab.med.yale.edu/modeldb/, acc.n. 266910).

### Simulations

For all simulations, we used networks of 550 neurons (500 excitatory and 50 inhibitory) connected using four different topologies: an exponential connectivity rule, with or without a distance-dependent connection (ER and ER-dist models), or a convolutive model reproducing the in- and out-degree distributions experimentally observed in a *C. elegans* brain^22^ or in a mouse hippocampus slice^23^. For simulations where the connection length distribution was also considered, neurons were randomly distributed in a 3D volume shaped either as a rectangular 500 × 500 × 2000 μm volume (for *C. elegans* connectivity), or as a 400 × 300 × 10 μm slice (for hippocampal connectivity). All simulations were implemented using Matlab^44^, and model files will be available on ModelDB (acc. n.266910). Following a custom practice for this type of electroencephalographic (EEG) experimental recordings, simulation traces were filtered with high-pass (1 Hz) and a low-pass (40 Hz) Butterworth filters^14^. This allowed a better comparison between modeling and experimental results.

To activate the networks, inputs were implemented as external currents with a peak value located at the center of the model, and modulated according to the spatial soma locations as:

12 $$I_{i} \left( {t,m} \right) = S\left( t \right)\left( {I_{{max}} e^{{ - \frac{{m_{x} ^{2} + m_{y} ^{2} + m_{z} ^{2} }}{{2\sigma _{{xyz}} ^{2} }}}} + I_{{GND}} } \right) + \zeta _{i}$$

where {*mx*, *my*, *mz*} are the i-th soma coordinates, *I*_*max*_ = {1.5 nA, 2.5 nA, 5 nA}, *I*_*GND*_ = 1 nA, *S*(*t*) is the time-dependent component of the current, with values between 0 and 1, *σ*_*xyz*_ = 600 µm, and ζ_i_ a random normal vector with amplitude 0.1 nA.

It demonstrates that this type of model is able to reproduce epileptiform activity and spectral properties in good qualitative agreement with experiments. In order to have a quantitative measure of an epileptiform activity, we used the average network activity, calculated from the average potential of the neurons^10^ as:

13 $$X\left( t \right) = \frac{{\sum\nolimits_{{i = 1}}^{{N_{E} }} {x_{{Ei}} (t)} + \sum\nolimits_{{j = 1}}^{{N_{I} }} {x_{{Ij}} (t)} }}{{N_{E} + N_{I} }}$$
